# Application in environmental art design practice based on a fuzzy evaluation system

**DOI:** 10.1038/s41598-024-62477-2

**Published:** 2024-05-30

**Authors:** Yongliang Sang

**Affiliations:** College of Art and Design, Yancheng Teachers University, YanCheng, 224007 China

**Keywords:** Environmental art design, Fuzzy evaluation system, Decision-making, Technique for order preference by similarity to ideal solution, Visual appeal, Environmental impact, Environmental social sciences, Mathematics and computing

## Abstract

Environmental art design (EAD) has recently encouraged creative thinking by investigating novel materials, technologies, and techniques for designing environmental art that advances sustainability. EAD faces challenges in integrating novel materials and technologies while promoting sustainability. Environmental art design is targeted at human living areas; here, adequate and excessive utilization of resources is minimized, and the social and natural environments are utilized aesthetically. Aesthetic excellence in environmental art design, along with growing technological accomplishments and cultural heritage, is concentrated on meeting the demands of human aesthetic pursuits in the new era, which lacks earlier techniques. Hence, an algorithm named environmental art design using fuzzy (EADF) to evaluate the environmental criteria for better decision-making is introduced. Initially, a fuzzy-based technique for order preference similar to the ideal solution (FTOPSIS), which considers multiple variables such as visual appeal, environmental impact, sustainability, and audience involvement in the community, was employed in the design process. Environmental art designers utilize fuzzy TOPSIS to assess works of art using several criteria. It seeks to make accurate decisions and accomplish desirable creative effects by considering ambiguity and subjectivity. The approach utilizes fuzzy variable entropy analysis to determine questionable attribute weightings and employs triangular fuzzy numbers to represent criteria and analyze preference values. Artworks are evaluated for deviation from ideal results using the Euclidean distance measure, enabling logical ranking evaluation and comparison. The EADF model outperforms the other models when considering different input factors. EADF excels in color (83.74), composition (82.37), emotion (85.61), contrast (97.52), clarity (98.16), harmony (95.49), and sensitivity (96.44) when evaluated in environmental art design, showcasing its usefulness. This work has implications for directing artists, designers, and decision-makers toward environmentally sustainable and artistically impactful art practices. Hence, the performance of this EADF model is validated using audience involvement, environmental impact, sustainability, and a visual appeal score.

## Introduction

The design of real-life environments that primarily respond to individual behavioral demands and aesthetic desires based on environment–behavior associations can be called the art of environmental design. Environmental art design is a creative approach that concentrates on developing artwork, carvings, and designs that blend with and are inspired by nature. This type of art frequently aims to generate knowledge about issues related to the environment, build a stronger bond between humans and the environment, and promote sustainable processes. More environmentally friendly merchandise can be designed by addressing difficulties arising between environmental effects, expenses, and product performance. The critical process of constructing the experience is individuals’ perception of the environment related to a person’s experience, which is “the practical actions that occur and impact the emotions of others that may leave an impression”.

Structure and interest in the visual field should be considered using both fuzzy and complicated impact artistic assessments. Fuzzy theory is a reliable way of aggregating expert decisions and delivering consistent results, and fuzzy logic enables the depiction of inaccurate or ambiguous judgments that do not fit into rigorous number values. This project’s main objective is to develop a comprehensive evaluation methodology for Environmental Art Design (EAD) that considers evolving technology environments and public expectations. Its primary objective is to produce art that enhances people’s visual and sensory experiences with minimal resource demand, particularly in residential locations. Many aspects of EAD, including aesthetics, environmental effects, sustainability, and audience participation, are acknowledged in this study. The foundational approach is the fuzzy-based technique for order preference by similarity to the ideal solution (FTOPSIS).

This study is driven by the connection between environmental awareness, creative creativity, and the necessity for a structured method to assess artworks within environmental art design. The lack of research on this topic is due to the absence of a systematic methodology that fully incorporates aesthetic, environmental, and sustainability factors. This work fills a gap by introducing and applying FTOPSIS in environmental art design. The research gap in evaluating environmental art design is due to the absence of a thorough evaluation framework, restricted use of quantitative methods, and inadequate incorporation of sustainability metrics. Current research frequently emphasizes aesthetic or environmental factors while overlooking comprehensive assessments. Quantitative methods can be subjective and qualitative, impeding evidence-based decision-making in selecting and creating artwork. Sustainability is an important issue; however, its incorporation into art assessment methods is still restricted. This paper presents FTOPSIS, a decision-making method used to assess artworks in environmental art design. The process provides a systematic, structured, and quantitative way to evaluate artworks using aesthetic, environmental, and sustainability standards. The comprehensive evaluation methodology considers visual appeal, environmental effects, sustainability, and audience engagement. This study fills the research gap in fragmented art evaluation and advocates for eco-friendly practices in the artistic community. The FTOPSIS-based methodology assists artists in selecting materials and methods that align with aesthetic objectives and environmental sustainability.

This study builds on an examination of triangular fuzzy number scales, which are based on fuzzy set theory, to address human language. Collaborative decision-making is enhanced by the art design’s incorporation of linguistic word sets and uncertain fuzzy value linkages. The EADF method considers aesthetics, ecological influence, longevity, and consumer participation to evaluate environmental artworks. The fuzzy technique for order preference by similarity to the ideal solution (FTOPSIS) method simplifies the evaluation of environmental art projects. This study aims to make EADF (environmental art design) a better tool. Its efficacy is proven by employing performance measurements, the FTOPSIS method, a multicriteria evaluation, and the measurement of Euclidean distance. It also addresses subjectivity and ambiguity. The EADF algorithm is an effective decision-making tool for addressing the complexity of environmental art because of its comprehensive evaluation framework and capacity to adapt to changing technology and social situations, which contributes to the concept of EAD. The TOPSIS methodology was selected to effectively manage multiple criteria for decision-making difficulties. TOPSIS is known for its simplicity, resilience, and capacity to evaluate options based on their proximity to the optimum solution, considering several competing criteria. Its capacity to handle the intricate decision-making process involved in environmental art design—in which several factors must be harmonized to produce ideal results—makes it appropriate for this study.

The EADF algorithm is a game-changing resource for environmental art design evaluations and decisions. Its comprehensive multicriteria approach considers all aspects, including aesthetics, the ecological footprint, sustainability, and audience engagement. This technique uses triangular fuzzy numbers and fuzzy variable entropy analysis to address ambiguity and subjectivity. It is an invaluable tool for making decisions and helps with reasonable rankings and educated choices. The EADF model can be modified to accommodate changing cultural, technological, and creative contexts, and performance metrics provide credence. This new viewpoint provides practitioners with an advantage in the ever-changing field of environmental art design. Given the aesthetic qualities of design functions, environmental art design pleasures the body and thought, enhances life, and promotes the sustainability^1^ of living and designing the aesthetics that play a part in the execution of environmental art design. Computers have significantly changed our lives as science and technology have advanced. The application of computer-assisted design^2^ for environmental artistic design creation can break beyond traditional forms of expression and empower design thinking. Photoshop software can provide accurate and multiple dimension-based design impacts, display design purpose most transparently, and significantly improve the effectiveness of environmental design artwork^3^ in landscape and interior form. The successful integration of contemporary artwork design and folk art via virtual reality was imposed in Ref.^4^ to overcome the constraints associated with conventional environmental art design, providing artists with a multiangle approach and designing more compassionate and attractive environmental art design practices. To design sustainable products in interior spaces and maintain their environmental properties, such as lifecycle span, energy use, and maintenance cost^5^, this research applied the building information modeling (BIM) environmental design approach for decision-making. This study gathered and analyzed environmental art design knowledge and approaches to categorizing and interpreting existing data to create the database required for a virtual representation^6^ of the environmental art design. The application of AI has a long-term and imaginative impact on landscape collaboration^7^ in environmental artwork, dynamic setup, and digital sketching in a linear sequence on a multiple-dimensional basis. Creative platform-oriented digital design^8^ includes applying AI technology to certain software systems that consider users’ preferences for environmental art design, such as deep drawing and deer class designs, to create art that may produce interactions among humans and machines in knowledgeable systems and provides academic support. The fuzzy integral^9^ was used to calculate the weight and performance of enterprises such as museum art licencings with cultural value to address the multicriteria decision-making challenge. Environmental art design provides joy to the laborer’s mind through the aesthetic elements of design efforts; the use of this AI technology not only fits the technical and security needs of energy enterprises but also helps to improve their visual quality^10^ and minimizes the costs involved with the construction of the energy sector’s environmental design. Designers prioritize the aesthetic effect of visual appeal^11^ of cultural venues on viewers when developing them based on human factors; however, vision is never a single experience of perception. Vision is frequently accompanied by comprehensive attitudes such as observing, feeling, and contact, incorporating aspects of architecture such as dimension, style, shade and light, resources, vibrations, and the environment, both indoors and outside, and generating staggering visual knowledge^12^. In addition to these indoor and outdoor environmental artworks, art design focuses on the energy sector to maintain the sustainability of the economy and reduce energy usage costs with the help of environmental art design, which causes few environmental hazards. A quantitative evaluation method that utilizes a comprehensive examination of the color of the material structure, design, and other features combines indoor wireless connectivity designs and cloud-based information to increase the application impact of sustainable environmental resources^13–15^. In addition to sustainable features^16^, work focused on ecological principles and strategies, increasing the quality of urban environments and building an excellent community image is important. The architecture of the kindergarten, as well as its indoor and outdoor environments, has been renovated and redesigned. Today’s design practice and study are undergoing a major shift, focusing on the goal of thinking and acting rather than objects^17^. The evaluation of art design and its related attributes has revealed that a culture’s worth depends on its past and heterogeneity. Environmental art design naturally reinforces budget accuracy, improves the relationship between the two groups in the design process^18,19^, simplifies landscape exhibitions, and replaces the conventional situation in which the thinking expression of artists with 3D technology constrains environmental art design. The environmental art design artists select materials, organic assets, and artistic components such as texturing software and visual space allocation^20,21^. Fuzzy TOPSIS, a method for fuzzy preference ordering according to resemblance to ideal solutions, was used to assess environmental art concepts in this study. The challenge of art evaluation is overcoming subjective judgments and imprecision, and this approach does just that. In addition, it allows for comprehensive criterion evaluation that considers factors such as sustainability, aesthetics, community involvement, and ecological influence, among others. The method also employs a weighting technique to make nuanced grading easier. Using both positive and negative ideal solutions, Fuzzy TOPSIS offers a comprehensive decision-making approach that can be applied to environmental art design. It is a flexible approach for handling the intricacies of environmental art design, and it works with fuzzy logic.

The EADF algorithm uses the TOPSIS methodology, particularly in its fuzzy variant (FTOPSIS), to assess the impact of public artworks on the environment. Aesthetics, environmental impact, sustainability, and audience engagement can all be effectively managed with TOPSIS. It employs fuzzy logic to consider subjectivity and ambiguity, enabling a more thorough evaluation. FTOPSIS combines art and environmental science by offering a systematic method for assessing artworks using aesthetic, environmental, and sustainability standards. The review approach considers visual appeal, environmental effects, sustainability, and audience involvement, allowing for a comprehensive assessment beyond traditional aesthetics. FTOPSIS adds a quantitative aspect to art assessment, aiding in making decisions based on evidence in the creative process. It addresses the complexity and ambiguity of environmental art design by offering a formal framework for evaluating artworks using predetermined criteria and weights. FTOPSIS encourages sustainable art practices by integrating sustainability criteria into the evaluation process and advocating for utilizing eco-friendly materials and processes in art creation. Through metrics for both the ideal and negative ideal solutions, TOPSIS enables the systematic appraisal of artworks.

The major contributions of this work are as follows:

- Applying a comprehensive strategy that considers aesthetics, impact, sustainability, and community engagement brings art evaluation to a new level.
- Fuzzy logic is implemented in the evaluation process to capture the complexities of the aesthetics of the arts and their environment by producing a natural and diverse evaluation process.
- By empowering artists with a strong framework for making informed decisions, artists can balance artistic genius with beneficial effects in the ever-changing field of environmental art.
- A comparative analysis is performed to verify the proposed model’s efficiency using different environmental metrics.

The remainder of this paper is structured as follows. “Literature survey” section reviews the previous literature to identify the aspects and criteria influencing the evaluation of environmental art designs. “Proposed system” section describes the research methodology using fuzzy-based evaluation in environmental art design practices. “Results and discussion” section provides numerical illustrations of the proposed approach and a comparison with existing techniques. Finally, the discussion and conclusions are presented in “Conclusion” section, along with future research directions.

## Literature survey

Singh et al. proposed an approach based on the fuzzy analytical hierarchy process and the technique for order preference by similarity to ideal solution (FAHP-TOPSIS) to prioritize the alternatives that surmount the constraints in eco-design practice in small and medium enterprises (SMEs)^22^. Their results showed that the sensitivity of the analysis was 0.60, and the bias in the outcomes due to experts’ and other countries’ perspectives on eco-design implementation may differ slightly.

Wei and Madina focused on using environmentally friendly materials in kid furniture design, integrating fuzzy with structured (F-S) technology for design to create a fuzzy technology-based furniture design system for children and two intelligent furniture design systems for children^23^. This research highlighted environmentally friendly children’s furniture design; nevertheless, it did not include exploring alternatives, analyzing user experience, or fully assessing environmental impact.

Xu et al. proposed an art design expertise communication teaching and practice centered on the modern multimedia environment to create teaching expertise and techniques. Four-dimensional goals for teaching, five criteria, and six tiers of IG-GC were used on the symbolic basis of the multimedia environmental art development principle, as well as the current teaching situation in China^24^. A major research gap was that this study focused only on the origin level of environmental art design.

Zhu et al. evaluated the visual impact of the artistic design of environmental schemes via a hybridized analysis of 4 multiattribute decision-making systems (H4MADM) that incorporated the fuzzy Delphi method (FDM), exploratory factor analysis (EFA), the analytic hierarchy process (AHP), and a decision-making trial and evaluation laboratory (DEMATEL)^25^. A major research gap that was identified was the lack of focus on the built setting in decision-making.

Xu et al. developed the hybridized conformal prediction algorithm (HCPA) model to handle the characteristics of indoor design, such as the client-oriented plan, environmental coordination, rational, safety, and artistic space of environmental art design with the incorporation of sustainable products^26^. The framework achieved 98% design correctness, 92% design execution, and a 6% error rate.

Guo et al. provided a community museum excellence assessment method that utilizes principles of human-centered design through the viewpoint of community-based museums and demonstrated the application of the computational concept of the fuzzy comprehensive evaluation technique to communal museums (FCET-CM)^27^. Due to time and resource constraints, this study focused solely on the level of assessment of community-based museums.

Zhang contrasted the image of environmental art design produced through AI with an image of traditional manual sketching^28^. The designer’s use efficiency increased dramatically by 84%, and the three-dimensional effect of the drawing surface was enhanced with improved quality. The factors considered for drawing were clarity, completeness, stereopsis, and frames per second, with a detection range of up to 120 min with a 20-min interval.

Xie et al. provided a full-connection strategy and greedy technique for assessing the environmental effects of urban environments that involved visual and semantic evaluation using high-dimensional attributes of public art design^29^. Their results showed that the audience and producer precision of public art design service areas were 0.87% and 0.78%, respectively.

Hou and Xu proposed AI in environmental design to analyze influential design works and evaluated the value and importance of artificial intelligence in contemporary environmental design, along with its possible long-term growth pattern^30^. Research gaps included a lack of specificity, an inadequate discussion of content transformation, an inadequate evaluation of the evolution of design languages, and an in-depth analysis of influential works.

Agarwal et al.^31^ introduced the probability-based double uncertain fuzzy (PDHF) algorithm as a novel approach for making group decisions involving multiple attributes in uncertain fuzzy settings. It creates a thorough decision evaluation matrix when used alongside the preference ranking organization method (PROM).

Garg et al.^32^ presented a novel multiattribute decision-making (MADM) method for intricate and unpredictable settings. The data representation involves the use of bipolar fuzzy information and Aczel-Alsina operators. Additional research is needed, which involves validation, robustness analysis, comparison with existing approaches, and stakeholder feedback.

Based on a literature review, scholars have addressed eco-design issues and impediments well. Nevertheless, there is room to update the challenges faced, such as weighting procedures, quality approval, outcome bias, and time and resource constraints; hence, there is a need to focus on the application criteria in environmental art design practice. As a result, there is a need to recognize and evaluate the hurdles to eco-design applications in an environmentally friendly manner. Making decisions in environmental design using the FTOPSIS method is systematic, considers various factors, and minimizes prejudice. It has the potential to fill knowledge gaps in areas such as digital technology’s effects on art and the environment, eco-design results, AI integration into the user experience, and AI in modern design. Deep analysis, longitudinal investigations, and comparisons of different approaches are all areas that benefit from this approach as well. Algorithms that were used in this comparison study include FAHP-TOPSIS^22^, H4MADM^25^, and FCET-CM^27^.

## Proposed system

The use of fuzzy logic in evaluation systems has been actively researched due to its flexibility and dependability. Linguistic data can be handled more easily and conveniently. The artwork relates interior and exterior environments to reflect a particular environment and style to satisfy people’s practical and visual aesthetic demands. The fuzzy evaluation method is based on breaking down an assessment problem into several subcriteria, evaluating each subcriterion, and eventually calculating the overall score of the basic evaluation of environmental art design practice based on the cumulative weight calculation. The goal of the EADF is to enrich the attractiveness of an environmental space through strategic art design while constantly serving the practical demands of humans. The purpose of environmental art design is to create an exquisite experience of time in the space environment, whether in the interior or exterior. Environmental art design is evaluated using a variety of factors, such as artistic inventiveness, environmental effects, cultural relevance, social participation, sustainable adoption of sustainable materials, and longevity. Each of these factors contributes to the initiative’s ultimate achievement and efficiency. These could entail developing a complete decision-making approach that includes qualitative and quantitative factors.

Higher standards are being set for environmental art specialists because of contemporary computer science advances. FTOPSIS calculates the distance between each alternative and the most and least efficient solutions for each criterion. This method helps evaluate environmental art designs that involve multicriteria decision issues because it incorporates fuzzy logic, which accounts for the inherent imprecision in human judgments. Combining disparities across many attributes into a single measure, such as geometrical distance, makes it possible to evaluate the overall performance of each artwork using FTOPSIS, which is useful for analyzing artwork design based on multiple criteria. The EADF algorithm uses fuzzy logic to address uncertainty in aesthetic ratings. It incorporates FTOPSIS, a fuzzy-based method, to model fuzziness and imprecision in art evaluation. Environmental art design can be evaluated using FTOPSIS, a robust MCDM methodology based on various criteria, including aesthetic appeal, environmental impact, sustainability, and audience involvement. It uses triangular fuzzy integers to represent imprecise information and discovers unknown attribute weightings. Utilizing the Euclidean distance measure, the EADF assesses the extent to which potential solutions depart from optimal ones. The FTOPSIS provides a comprehensive evaluation for decision-making.

The FTOPSIS model is a systematic way of assessing artworks in environmental art design. The process includes identifying evaluation criteria, standardizing them, establishing weighted criteria, and developing fuzzy sets to reflect linguistic variables. A decision matrix is created by applying weights to normalized criteria for comparison with predetermined criteria. FTOPSIS calculates the similarity of each alternative to the ideal solution by assessing their proximity to the positive and negative perfect solutions. Alternatives are rated based on their proximity to the positive ideal solution and distance from the negative ideal solution, with closer options being preferred. The last step consists of assessing and contrasting the rated options according to their closeness to the perfect solutions, determining the most appropriate artworks based on aesthetic appeal, environmental effect, sustainability, and audience engagement. This strategy encourages the incorporation of aesthetic, environmental, and sustainability factors in art production and assessment, aiding in well-informed decision-making.

**Figure 1:**
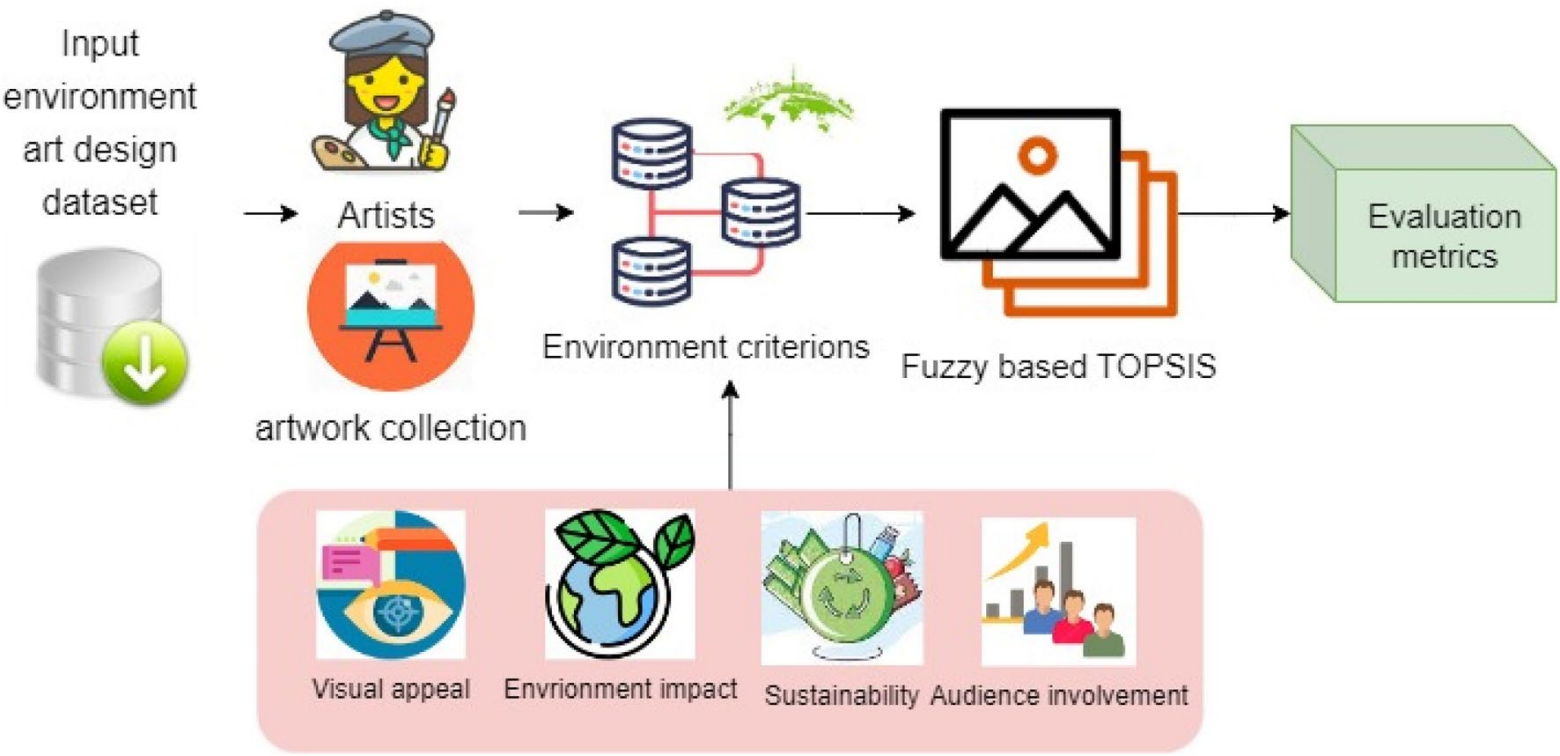
Overall architecture of the proposed EADF system.

This study focused on environmental art design with fuzzy (EADF) technology, a new paradigm for evaluating environmental artworks. The EADF approach has been beneficial for assessing art projects in various contexts, including those involving sustainability, community engagement, visual appeal, and environmental impacts. The EADF methodology utilizes fuzzy logic and fuzzy TOPSIS, a fuzzy technique for ranking alternatives according to how close they are to an ideal solution, making it easier than ever to assess environmental art proposals. The model provides a more all-encompassing picture of the artwork’s efficacy since it considers many different things. The EADF model ensures a good decision-making process by allowing art to be rated and chosen congruently with specific environmental and aesthetic goals. The fuzzy TOPSIS approach achieves optimal outcomes by establishing criteria for standardization, finding the entropy for weight computation, computing a weight-normalized fuzzy relationship matrix, using Euclidean distances, and assessing relative proximity. Figure 1 presents the overall procedure of the proposed algorithm for evaluating environmental art design based on fuzzy TOPSIS.

Initially, the input dataset for environmental art design was collected from open-access museums with two major attributes focused on artists and artwork. The artist’s attribute consists of individual identifiers with additional information, and the artwork collection has many records, such as title, credits, dimension, acquisition date, material used, and environmental impacts. For evaluating the environment, criteria related to art design were considered for visual appeal, sustainability, audience involvement, and environmental impact. The evaluation was performed by implementing an FTOPSIS algorithm followed by performance metrics for evaluating the environmental art design and suggesting decisions for audiences related to the art design practice that supports all the environmental aspects. In environmental art design, fuzzy TOPSIS is a popular tool for making multicriteria decisions. Accurate measurements are made possible by the efficient management of subjective and unpredictable parts of artwork judgment. To account for the variety in subjective aesthetic evaluations, fuzzy TOPSIS additionally converts language variables to fuzzy numbers. It is a good option for detailed assessments due to its comparable effectiveness in addressing inaccurate data. The approach aligns with the study’s goals, which were to conduct an all-encompassing evaluation of artworks considering their aesthetic value, ecological footprint, sustainability, and audience participation.

### Step 1: evaluation of the criteria for environmental art design

The initial process defines the main criteria for evaluating environmental art design practice decisions, including (i) visual appeal, which refers to an artwork’s or design’s aesthetic appeal and attractive aspects. The structure, color balance, harmony, contrast, uniformity, and dimensions such as length, height, and width, in addition to the overall appearance, are all included. A piece of artwork with strong and visually appealing qualities is more likely to attract visitors and leave them with a pleasant initial response with second-level indicators such as department and classification, such as printed books, illustrations, photography, sculpture, drawing, painting, video, film, paperwork, Fluxus collection, architecture and design^33^. Through the visual qualities of the work, aesthetic appeal grabs the audience’s attention and interests. Environmental art design factors that influence (ii) environmental impact include material selection, location, the possibility of disturbing processes of nature, and the enduring viability of the art piece. (iii) Community engagement refers to the involvement of collaboration with residents, credits, media of art design for the entire process of art planning, the experience of artists, and the credit they achieved. (iv) Sustainability is focused on reducing negative environmental impacts through the use of natural, eco-friendly art design materials such as cotton, ink, paper, and wood; preserving resources; and avoiding the use of hazardous materials.

The evaluation set and fuzzy values needed to be created to identify criteria in environmental art design include visual appeal (c1), environmental impact (c2), community engagement (c3), and sustainability (c4), followed by the assignment of each factor with linguistic values as follows. c1: {high, medium, low}, c2: {strong, moderate, mild}, c3: {active, partial, passive}, c4: {very much, average, less} with alternatives α as artists ${\alpha }_{1}$ and artwork-based ${\alpha }_{2}$ evaluation. The fuzzy membership function is applied, and the corresponding linguistic concepts can be translated to fuzzy integers. Triangular fuzzy integers represent each of the criterion variables in the decision matrix.

**Figure 2:**
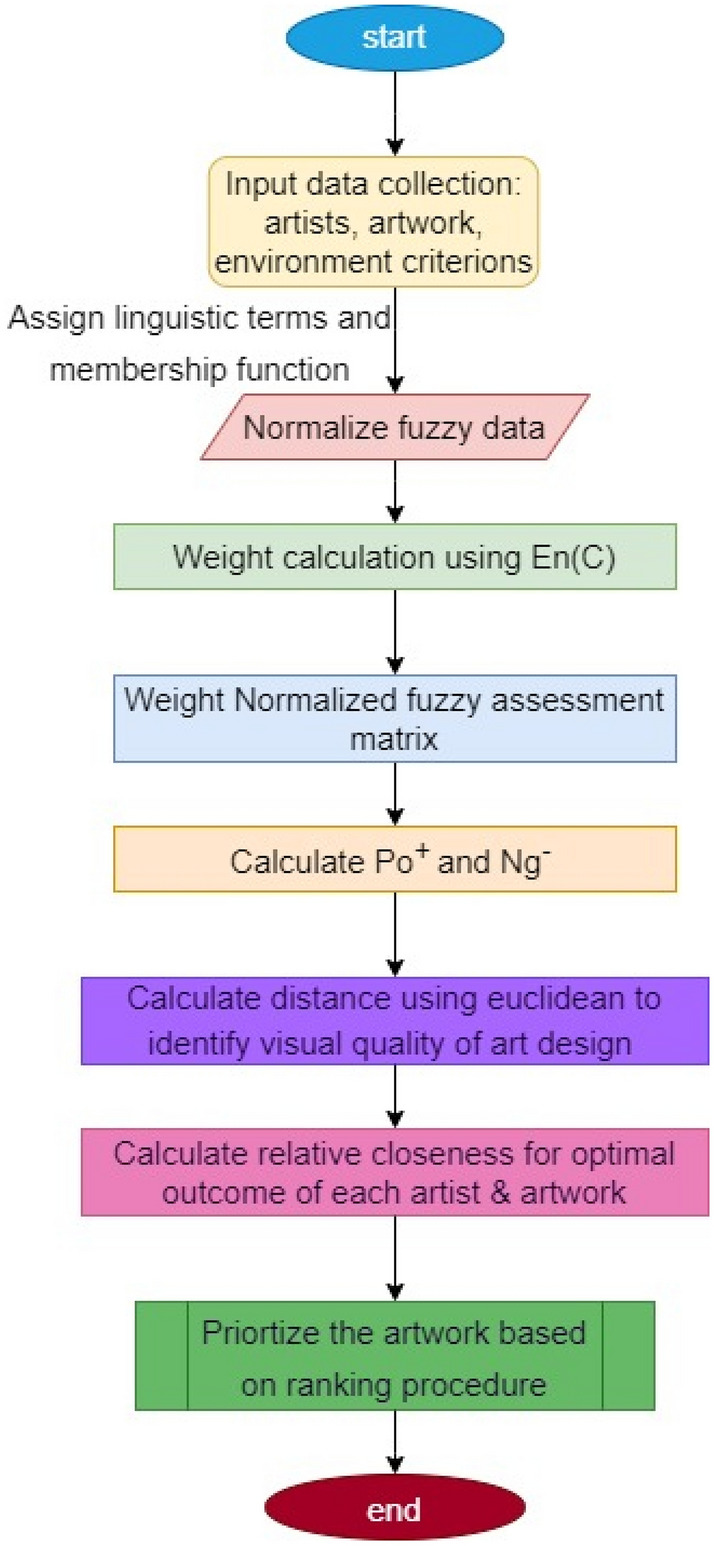
Flow chart of FTOPSIS.

EAD incorporates aesthetic and qualitative characteristics that may not be perfectly quantitative; fuzzy numbers are an appropriate approach for representing these subjective judgments. For example, visual appeal and community engagement are EAD criteria that may be interpreted using triangular fuzzy numbers that provide a robust technique to integrate subjective judgments, uncertainties, and qualitative features into art design evaluation. They serve as a link that connects artistic and quantitative components, facilitating a more integrative and appropriate evaluation of artwork performance across multiple parameters. As a result of solving the mentioned model, a simple formula for evaluating attribute weights was produced. Figure 2 shows the flowchart representation of the fuzzy-based TOPSIS evaluation technique for environmental art design.

The subject of this research is the method for converting conceptual ideas from language into fuzzy numerical values for use in environmental art design evaluation. Assigning fuzzy numbers to each variable, classifying them into fuzzy sets based on expert input, and using adaptive transformations to account for different interpretations among evaluators were all part of the process. The effect of different fuzzy set definitions on the final assessment outcomes was investigated using a sensitivity analysis. This systematic and adaptable methodology ensured a thorough and reliable evaluation of artworks in environmental art design, bolstering the scientific rigor and trustworthiness of the evaluation process. The study’s methodology is crucial since aesthetic evaluations are subjective and open to different interpretations.

### Step 2: normalizing the criteria for fuzzy data

Using the normalized fuzzy matrix ${\uplambda }_{i}$, the values of each criterion are scaled to a common scale, generally approximately 0 or 1. This matrix comprises the fuzzy assessments of options concerning each criterion in the environment of multicriteria decision-making. Normalizing fuzzy data entails converting linguistic evaluations or fuzzier numbers to similar values that can be employed in calculations such as the TOPSIS method. Normalization is carried out in this step of the TOPSIS technique to provide consistency in criteria and equitable impact on decision-making. The decision matrix is normalized using the vector normalization approach, which involves dividing every component by the square root of the sum of squares of all entries in the respective column. The normalized decision matrix can be calculated as

1 $${f}_{ij}=\frac{{p}_{ij}}{\sqrt{\sum {\left({p}_{ij}\right)}^{2}}}\text{ for }i=1\,to\,m\text{ and }j=1\,to\,n,$$

where ${f}_{ij}$ represents the normalization score of fuzzy matrices with the *j*th criterion and for the *i*th alternative in Eq. (1).

Normalizing fuzzy data, finding entropy for each criterion, calculating the weight-normalized fuzzy relationship matrix, finding positive and negative optimal solutions, computing distances using Euclidean distance, and calculating the relative closeness for optimal outcomes are all steps in the structured process known as the fuzzy TOPSIS method. To standardize linguistic assessments or fuzzy numbers into a common scale, the first step is to normalize the fuzzy data using a fuzzy matrix (${\uplambda }_{\text{i}}$). Each criterion’s impact on environmental art design can be better understood using entropy calculations for weight calculations, which helps evaluate their uncertainty and distinctness. Multiplying the normalized criterion scores by their corresponding weights integrates the relative importance of each criterion and forms the weight-normalized fuzzy relationship matrix. Distances measured in terms of standard geometrical units allow us to compare and contrast works of art according to several parameters. To gain insight into the performance of artworks in terms of aesthetic appeal, environmental effects, sustainability, and audience involvement, the relative closeness calculation analyzes how close they are to ideal or least optimal outcomes.

Normalization eliminates biases caused by differing measuring scales and allows you to weigh every criterion according to its importance rather than the original assessment ranges. After the data have been normalized, FTOPSIS can be used to determine metrics such as reliable relative distance factors. The result assures that the assessment process is impartial, precise, and indicative of each criterion’s actual contributions to the overall success of the artwork design.

2 $${\uplambda }_{i}={\left[{f}_{ij}\right]}_{n\times z},$$

where ${f}_{ij}$ represents the normalization score of fuzzy matrices in Eq. (2), $i=\text{1,2},..n$ represents the raw value of the metric from the artists’ artwork attribute, and $j=\text{1,2},..z$ represents the minimum possible value of the total attribute.

### Step 3: determining the entropy for each criterion for weight calculation

A fuzzy entropy metric splits the input feature space into decision areas and identifies appropriate traits with adequate distinctness for the classification job. The proposed fuzzy entropy-based feature selection method improves the classification rate by removing noisy, ineffective, and irrelevant information. To capture the viewer’s attention, the uncertainty of the design system is used for calculating indicator weights to elucidate the impact of various indicators on environmental art design. Additionally, the attributes are ranked in an environmentally friendly order to quantify the extent of variation or dispersion in the normalized results for each criterion. The smaller the entropy and the more significant the weight the criteria should receive are, the greater the degree of consistency of the normalized results, as described in Eq. (3).

3 $$En\left(C\right)=-\sum_{i=1}^{n} {p}_{i}\text{log}\left({p}_{i}\right),$$

where $En\left(C\right)$ is the entropy of all the criteria considered in environmental art design with the probability distribution given as $P({C=c}_{i})$, where $n$ ranges from 1 to n. ${p}_{i}$ gives the normalized values of the criteria. The suggested method uses entropy analysis to derive the attribute weights of artwork alternatives ${\alpha }_{2}$ when the feature quantities are unknown. Triangular fuzzy integers represent the variables in the decision matrix used to arrange the linguistic term of each attribute with the specified criterion. The entropy values can be used to calculate the criteria weights, with lower values denoting more uniformity and knowledge. The fuzzy entropy weight of all considered criteria is calculated using Eq. (4):

4 $${w}_{c}=\frac{1+\sum_{i=1}^{n} {p}_{i}\text{log}({p}_{i})}{\sum_{j=1}^{n}\left(1-En(j)\right)},$$

where ${w}_{c}$ represents the weight of criterion $C$, $p(i)$ represents the probability, $E(j)$ is the entropy value, and $n$ is the total number of criteria being considered.

### Step 4: calculating the weight-normalized fuzzy relationship matrix

Each normalized criterion score ${f}_{ij}$ is multiplied by its appropriate weight, $W,$ to construct the weighted normalized matrix. For a successful outcome and accurate results, the impact of each element is considered together in a fuzzy assessment matrix. Triangular fuzzy integers represent each criterion variable in the decision matrix and offer alternative performance data on numerous categories, taking fuzziness or ambiguity into account. Let ${C=(c}_{1},{c}_{2,\dots .,}{c}_{i})$ be the set of criteria or attributes considered for environmental art design and $W={(w}_{1,}{w}_{2,\dots .,}{w}_{i})$ represent the weight vectors calculated from the entropy measures such that ${w}_{i}$ denotes the exact numerical integer and gives 1∀ $\sum_{i=1}^{n} ({w}_{i})\ge 0$. ${p}_{ij}$ is a fuzzy multiattribute decision-making problem that can be expressed in a matrix form called a fuzzy decision matrix $P={\left({p}_{ij}\right)}_{n\times z}\epsilon [\text{0,1}]$, as shown in Eq. (5).

5 $$\left[\begin{array}{ccc}{p}_{11}& {p}_{12}\dots & {p}_{1z}\\ {p}_{21}& {p}_{22}\dots & {p}_{2z}\\ ..& ..& ..\\ {p}_{n1}& {p}_{n2}\dots & {p}_{nz}\end{array}\right].$$

The horizontal and vertical representation of the matrix denotes the criteria and alternatives α (artists, artworks). The ${\left({p}_{ij}\right)}_{n\times z}$ decision matrix needs to be standardized in the form of each element in the matrix using the membership function.

Including the relative importance $RI$ of each element weighted in a single variable assessment matrix allows the aggregate impact caused by every element to be expressed understandably. $RI$ defines the relative importance value accessed in addition to the audience involvement score ${AI}_{s}$ calculated using Eq. (6); where, $s$ represents the score at which the artwork size compares with the remaining sizes with the assistance of the artwork ID.

6 $${AI}_{s}={(w}_{1}\times {c}_{1}(s))+ {(w}_{2}\times {c}_{2}(s))+\cdots +{(w}_{i}\times {c}_{i}(s))+(w(s)\times RI(s))$$

Design is a conceptually inventive activity where artists should become public leaders or guides, guiding the growth of trends and creating art design models in exhibitions, museums, and tourist places. Each artist has a separate ability to think about each environmental theme based on their creativity. The design practice of environmental art has audience coverage and requires that every artist have forward-thinking original concepts and an innovative, dynamic vision. An effective design must be creative since design involves the process of solving imaginative issues. The most important aspect of successful design is inventiveness. Following Eq. (7), to calculate the environmental impact, ${\Psi }^{i}$ is calculated using the corresponding input attributes of the artwork, such as the material, size, display, medium, indoor and outdoor space. The ${\Psi }^{i}$ is supported by the aggregated weight score of all the attributes with the normalized score ${\uplambda }_{i}$ that alters each single environmental design impact attribute.

7 $${\Psi }^{i}=\sum_{i=1}^{n}W{\uplambda }_{i}.$$

### Step 5: determining the positive and negative optimal solutions for each alternative

This supports the choice of artworks that are compatible with sustainable and visual objectives, allowing for a fair evaluation of options based on many criteria, such as visual appeal, environmental effects ${\Psi }^{i}$, and audience involvement ${AI}_{s}$. For each criterion $C,$, the maximum ${\uplambda }_{i}$ is identified across all alternatives α.

8 $${Po}^{+}={\text{max}}\left({\uplambda }_{ij} \forall {\alpha }_{j}\right),$$

9 $${Ng}^{-}={\text{min}}\left({\uplambda }_{ij} \forall {\alpha }_{j}\right).$$

Equations (8) and (9) explain the positive ${Po}^{+}$ and negative ${Ng}^{-}$ ideal solutions for criterion C, respectively. The normalized value ${\uplambda }_{ij}$ of alternative ${\alpha }_{j}$ is identified for each $i$ of criterion $C$ considered in Step 1.

The responses indicate the most beneficial and lowest potential results across the analyzed artworks for each criterion. These outcomes are subsequently utilized in the following steps, including distance calculation and ranking procedures, to analyze how every artwork operates concerning these criteria. Artworks closer to the ideal outcome perform better, while those further away from the unfavorable ideal solution do better. Based on numerous criteria, this technique supports objective assessment and informed decision-making conditions in environmental art design.

In Step 4, the fuzzy relationship matrix is normalized by adding weight information. This ensures that each criterion’s influence is proportional to its weight. Consistent with the multicriteria characteristic of assessments, this boosts precision by providing a more true representation of connections between artworks across numerous criteria. It sets the stage for a comprehensive review based on established criteria, such as visual appeal, environmental impact, expected lifespan, and audience engagement. A weighted matrix that incorporates the fuzzy relationship matrix (FRM) and the weights assigned to each criterion is called the normalized fuzzy relationship matrix (NFRM). The row index (RI(s)) is used to find the normalized fuzzy positive ideal solution (NFPIS), which accounts for positive ideal values and weights. Connecting the NFPIS to Eq. (6) allows us to calculate the separation measures for each choice, factoring in the weights and ascertaining the degree to which each artwork approaches the positive ideal solution. Steps 4 and 5 of the thorough evaluation are related. Step 5 uses the normalized fuzzy positive ideal solution (NFPIS), which is formed after Step 4 and creates the weight-normalized fuzzy connection matrix. The following steps involve exhaustively evaluating artworks using the data collected in Step 4.

### Step 6: calculating distances using the Euclidean distance

The distance of attribute values can be calculated using the Euclidean distance, which helps when comparing artworks. Attractiveness, community involvement, environmental effects, and sustainability analyses are all possible traits in this category. A smaller Euclidean distance indicates a greater degree of similarity, whereas a larger distance indicates a greater degree of dissimilarity. Its purpose is to compare the quality of different artwork designs to draw conclusions about how well they stack up against predetermined criteria. The environmental art design process involves determining the geometrical distances between each artwork and two sets of ideals and deficiencies, denoted as ${Po}^{+}$ and $N{g}^{-}$, respectively, from Eqs. (10) and (11). The resulting product is useful for rating and making well-rounded decisions, combining aesthetic vision and environmental objectives. The calculated distances show how each option fits the initiative’s needs and objectives. In terms of environmental art design, the solution aids decision-makers in selecting options that meet their desired criteria to the greatest extent.

10 $${d}_{{Po}^{+}}=\sqrt{{\sum }_{i=1}^{n}{\left({{w}_{i}(\uplambda }_{ij}-{Po}^{+}(i))\right)}^{2},}$$

11 $${d}_{{Ng}^{-}}=\sqrt{{\sum }_{i=1}^{n}{\left({{w}_{i}(\uplambda }_{ij}-{Ng}^{-}(i))\right)}^{2},}$$

where ${d}_{{Po}^{+}}$ and ${d}_{{Ng}^{-}}$ represent the Euclidean distance of the alternative from ${Po}^{+}$. The weight assigned to $C$ is represented as ${w}_{i}$ with $n$ number of criteria in the artwork. ${\uplambda }_{ij}$ represents the normalized value of alternative ${\alpha }_{j}$ with respect to each $C$ ranging from 1 to n. Calculating such distances allows the artist to determine the distance in each possible outcome from a positive and a negative ideal response in multiple dimensions. Considering the provided criteria and weights helps evaluate each alternative’s efficiency. Artwork appraisal involves stakeholders such as artists, curators, environmentalists, and community representatives. Artists and designers produce artwork and play a crucial role in comprehending its influence on people’s surroundings and viewers. Curators and officials from art institutions prioritize criteria based on public appeal, artistic merit, and subject relevance. Environmentalists and sustainability specialists evaluate artworks by considering their environmental footprint, utilization of sustainable resources, and adherence to eco-friendly methods. Audience and community representatives assess artworks, particularly in interactive art projects or public installations. The number of decision-makers participating in the assessment can fluctuate based on the circumstances and goals. Sometimes, one person or a group of experts may be in charge, while other times, a collaborative method with several stakeholders may be used.

### Step 7: calculating the relative closeness for the optimal outcome

In environmental art design, the mathematical calculation of relative closeness ${\mathbb{R}}$ using Eq. (12) analyzes artwork closeness, whether it is an ideal or least optimal outcome, by considering both artist and artwork features; hence, it can also be utilized to calculate the performance score of the artwork. It produces an impartial ranking, integrates many factors, and leads decisions by considering artistic and environmental concerns. It facilitates the selection of artworks that balance artistic intention with environmental impact. A higher relative proximity quantity implies that the performance of a possible outcome is comparatively close to that of the optimal solution. In environmental art design, this solution implies that the artwork performs well on the selected environmental criteria, such as visual appeal, sustainability analysis, environmental impact, and audience engagement.

12 $${\mathbb{R}}=\frac{{d}_{{Ng}^{-}}}{{(d}_{{Po}^{+}}+{d}_{{Ng}^{-}})}.$$

Environmental art design balances multiple goals: sustainability, environmental impact, visual appeal, and audience involvement. The calculation considers both elements and provides a full assessment by considering the distance relative to the negative optimal outcome. The derived closeness scores regulate performance evaluations, allowing for objective artwork ratings. Artworks with higher proximity scores are rated higher, indicating that they performed better on the overall criteria. The identified score is used to calculate the visual appeal score related to the input color attributes and the appropriateness, vitality, and coherence of the color palette with the concept of the artwork. Rate pace, points of focus, and entire visual rhythm for the composition variable to evoke the emotion of the audience intensity and range can be scaled. The artwork element and message used for communication with the environment and human beings are determined by the clarity of the art design.

### Step 8: identifying the ranking procedures for prioritizing the artwork

The goal of ranking solutions in environmental art design is to focus on artworks based on relative closeness scores, allowing for an informed decision that matches project objectives. A higher ${\mathbb{R}}$ score provides a better performance than all remaining attribute scores, and the rank is arranged in the form of a higher value toward a lower ${\mathbb{R}}$ value, leading to the lowest rank order; thus, it determines the ranking of the artwork in the evaluated order. This technique chooses artworks that perform better across various criteria, such as sustainability, visual appeal, and audience involvement. Decision-makers may efficiently locate and pick artworks corresponding to project goals by giving rankings, enabling educated and rational decisions that account for aesthetic and environmental concerns. Per the given procedure, the ranking from the vector normalization TOPSIS method is ordered as c2 > c1 > c3 > c4. This ranking order is completely determined by the relative closeness values computed using the vector normalization approach in TOPSIS.

The evaluation method of the EADF considers both individual and group goals. Recognizing that appreciation of art is fundamentally subjective, the algorithm incorporates several viewpoints. The final score considers each candidate’s unique tastes and the group’s collective norms equally. These factors are aesthetic appeal, environmental impact, sustainability, and audience participation. Fuzzy logic and many dimensions enable the EADF to consider the wide variety of subjective opinions in art. The entropy weight method was chosen because of its ability to objectively assess the relative importance of evaluation criteria. The entropy weight method incorporates a degree of objectivity while recognizing personal preferences by considering the intrinsic information value of each criterion. The entropy weight method assesses the diversity and uncertainty of the criteria, which aids in making better decisions. This ensures that the relative importance of criteria is not based on subjective opinions and accounts for the inherently diverse evaluation context. The EADF algorithm uses fuzzy logic and the entropy weight technique to balance objectivity and subjectivity. The entropy weight method adds mechanical neutrality to the aesthetic evaluation process, while fuzzy logic considers nuanced and subjective aspects. This holistic strategy strengthens the review’s validity and reliability by including diverse perspectives and the need for a structured, data-based methodology.

Eco-Art Design with Fuzzy (EADF) is a methodology that aims to enhance the incorporation of subjective and qualitative evaluations into environmental art design. Aesthetics, environmental impact, community involvement, and sustainability-related linguistic notions are typically defined first when developing fuzzy sets to accompany criteria. A triangular fuzzy number represents each linguistic concept; this method offers a structured way to handle ambiguity and imprecision. Assigning numerical values to each criterion, representing high, medium, or low degrees, allows for a comprehensive evaluation of environmental art design. When applied to language values, fuzzy membership functions transform them into fuzzy integers, guaranteeing a systematic representation of qualitative judgments. The use of triangle fuzzy integers to represent each criterion variable in the decision matrix allows for robust integration of subjective judgments, uncertainties, and qualitative features into the assessment of art design. The results of various art design works were evaluated by examining their ranking for each environmental criterion. The evaluated results assist in identifying each artwork’s qualities, shortcomings, and places for improvement. The outcomes are analyzed, and the rankings are utilized to make intelligent decisions about selecting or enhancing the artworks. The summary of the proposed EADF algorithm showed the possible evaluation outcomes of environmental impacts, sustainability analysis based on both artists and artwork with long-term impact among the audience, and making their involvement score as high as possible by frequently evaluating the visual appeal scores, color palettes, harmony, composition, and clarity for enhanced art design. Additionally, the visual appeal score based on artwork attributes should be increased in an environmentally sustainable manner.

## Results and discussion

### Data source description

**Figure 3:**
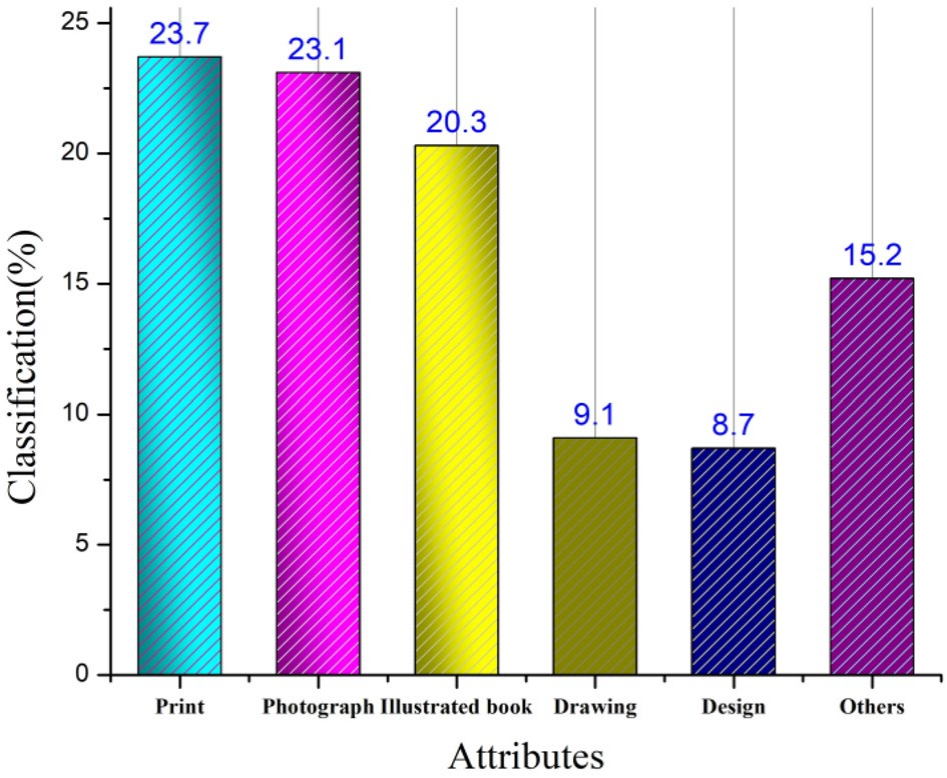
Classification of artworks.

The proposed model is analyzed using the dataset acquired from Ref.^33^, which includes data from the Museum of Modern Art, which includes 70,000 artworks from more than 20,000 art designers. Painting, the form of sculpture, the art of printing, sketching, imaging, design, construction, footage, forms of entertainment, and performance art are among the many forms of aesthetic artwork represented in the collection. The attributes related to this environmental art design source include the artwork ID, title, design medium (ink, paint, color (black, medium gray, very green yellow, very deep purple red, deep blue, and deep red‒brown), or graphite), art dimension (cm), and object number, in addition to the records of the artist’s identity (ID), name, nationality, and gender. The datatype representation is in integer form, and categorical representations are in .csv format. The main alternatives to attributes are artists and artwork as a key representation. Figure 3 depicts the classification of the artwork represented in the data sources.

### Evaluation metrics of the environmental art design

To determine whether the proposed approach is excellent and how it works, it is compared with existing methods such as FAHP-TOPSIS^22^, H4MADM^25^, and FCET-CM^27^ by considering evaluation metrics such as audience involvement on the basis of two variants, artwork and artists, the environmental impact ratio, sustainability analysis, and the visual appeal score.

### Audience involvement analysis

The attribute called the dimension of artwork is denoted on a horizontal axis that represents the degree to which the piece of art has been enhanced and enriched with different levels of importance calculated based on the proposed algorithm weighted by $En(C)$ and ${w}_{c}$, imagery, visual level of detail, height, and width of the art sheet in terms of inches and centimeters (cm) that improve its general level of quality. The level of audience involvement and impact is represented on the vertical axis. Engagement may involve a sense of connection, stimulating thought, and the artwork’s long-term impact on viewers.

**Figure 4:**
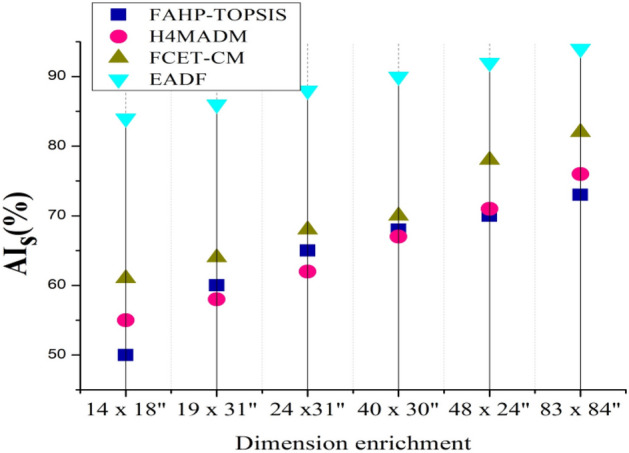
Analysis of audience involvement based on the artwork dimension.

**Figure 5:**
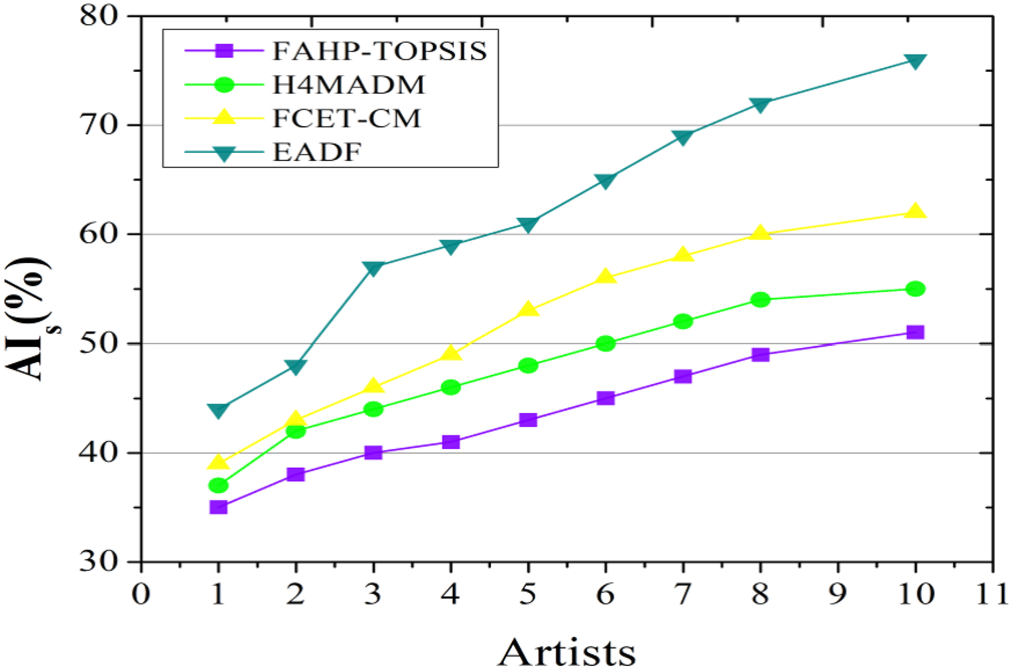
Analysis of audience involvement based on the artist’s alternative.

Figures 4 and 5 show that the artwork dimensions lead to a high outcome, positively impacting the audience’s involvement and increasing their attachment to environmental art practices. Knowing the proportions allows viewers to mentally put their concerns into the artwork, strengthening their relationships. They may evaluate how an artwork connects with the environment, regardless of whether it is a massive outdoor sculpture or a little internal sculpture. A large piece may elicit a different sense of emotion than a smaller piece, and viewers should consider how the dimensions add to the artist’s message.

### Environmental impact ratio

The input attributes of artwork include material usage, including plastic, painted wood, die cast, iron base, silver, acrylic, kindergarten, porcelain, stainless steel, rubber, resin, copper, fiber, glass, wire glass, building exhibitions, and eco-friendly cotton. The second attribute size includes site plan, white oak, sketches, natural-made trees, study model, housing, elevation, metropolitan, cemetery, plate size, mount size, book size, block, portfolio, aerial, and underwater. The third attribute display sample includes the fabric, floor, liquid crystal, leather, brushed steel, glass, cardboard box, metal, installation, beeswax, 2-way mirror, and micro LCD monitor, with intelligence. The fourth indoor attribute includes a fossil quarry mirror and tennis with outdoor artwork, including sculptures, mountains, a parking lot, a museum, and a theatre. The last attribute includes the medium of art design: wood, pencil, ink, collage, gauze, cotton cloth, and digital.

**Figure 6:**
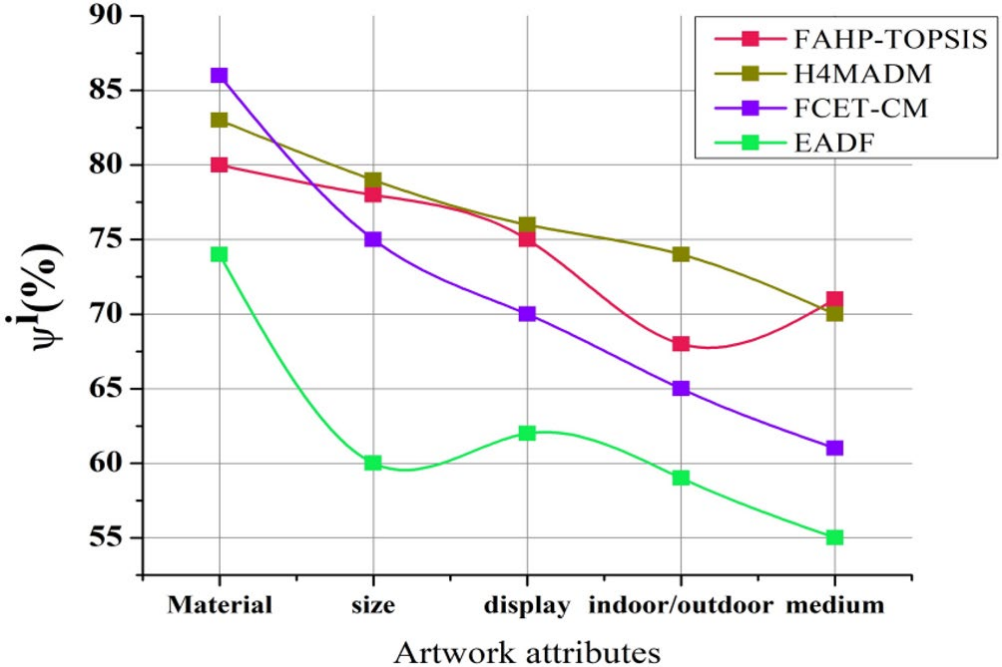
Environmental impact ratio.

Figure 6 depicts the environmental impact ratio; the analysis shows that a larger environmental impact ratio ${\Psi }^{i}$ generally suggests that the artwork or art design process under consideration has a greater negative environmental impact. In other words, a higher ratio indicates that the artwork’s aesthetic choices, materials, and other variables have a greater negative impact on the environment. The proposed EADF algorithm possesses a minimal amount of ${\Psi }^{i}$ compared to other existing approaches that use sustainable materials related to natural substances.

### Sustainability analysis

The duration and endurance of the materials employed can be utilized to assess sustainability in environmental artwork design. This measure evaluates the resilience and durability of the resources used in the artwork. It considers the degree to which the materials can survive environmental conditions and the passage of time. Durable materials may have less effect on the environment if they are replaced less frequently. This encourages artists to choose materials that add to the artwork’s long-term value and preservation while reducing the demand for periodic substitutes or repairs. The least duration is predicted in video game illustration, and the greatest infinite duration of the artwork remains when using the simulation form of design practices with linguistic variables of sustainability analysis (c3).

**Figure 7:**
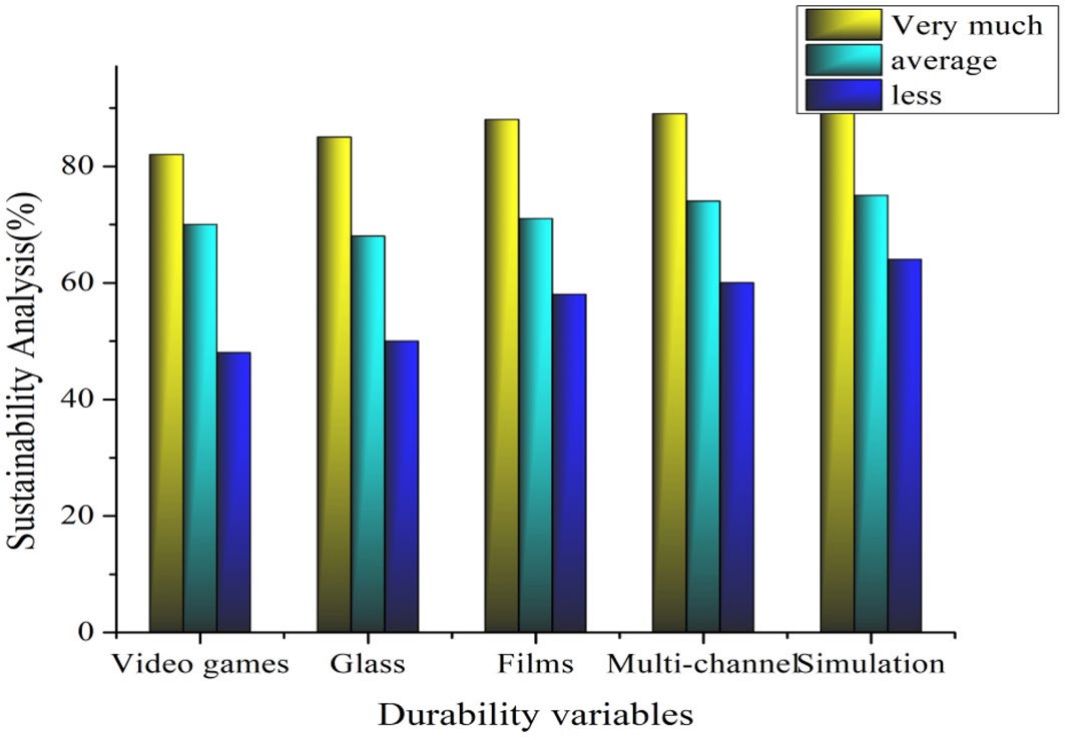
Sustainability analysis ratio based on the durability of the artwork.

**Figure 8:**
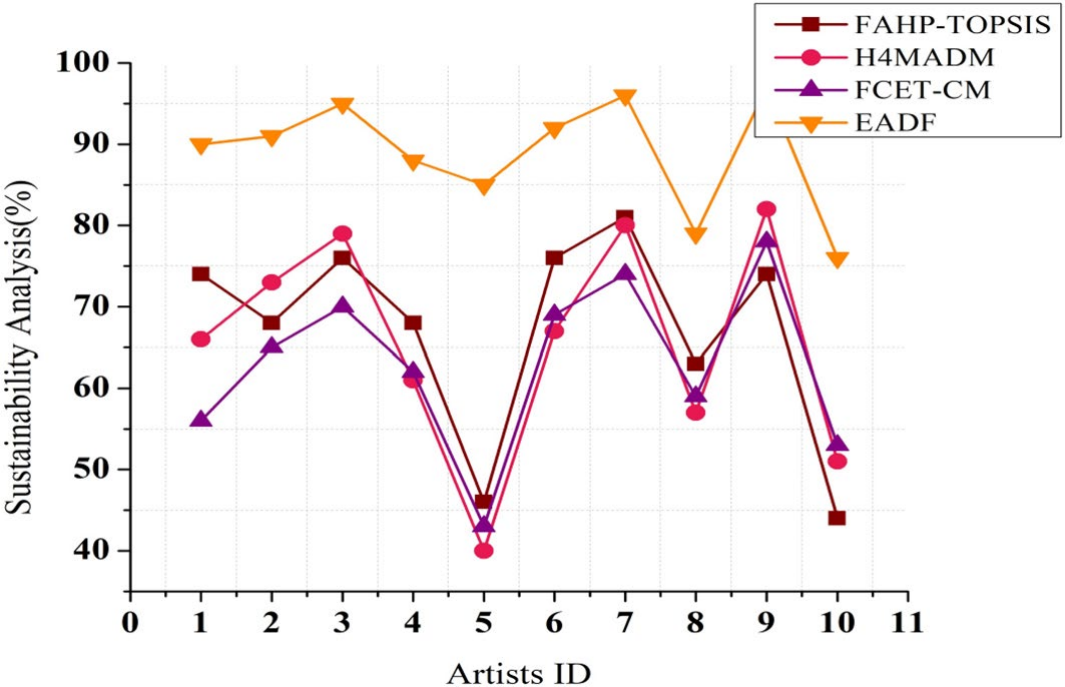
Sustainability analysis ratio based on the artist’s contribution.

The purpose of this work, as depicted in Figs. 7 and 8, is to build an artwork that can assist users in comprehending and successfully expressing the environmental effectiveness of various artworks. The life cycle of artwork, from birth through disposal, and its possible environmental impact are considered. The goal of determining the artist’s long-term impact on relevancy, influence, and capacity to inspire future generations in opposition to obtaining an overall sustainability score was analyzed, and the artist’s accomplishment was compared against these weighted parameters. The materials, classification, department of art creation, and environmental impact of each artwork related to the artist’s object identifier were examined.

### Calculation of the visual appeal score

**Figure 9:**
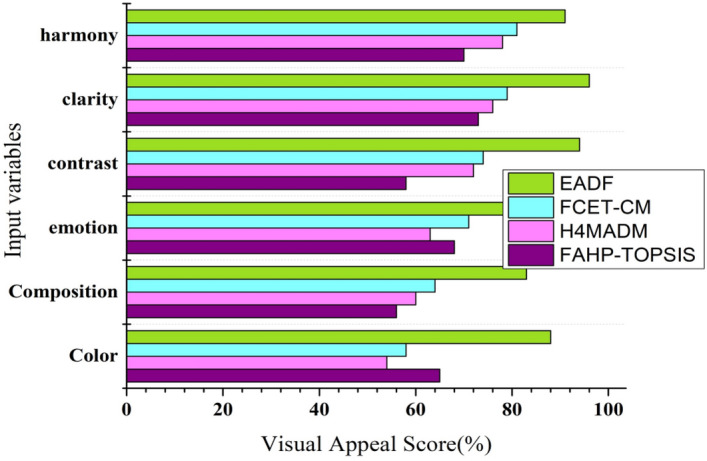
Analysis of visual appeal scores for artwork.

Figure 9 and Table 1 show the input attributes of artwork related to the criterion of visual appeal, including color, composition, emotion, contrast, clarity, and harmony. These are taken from the alternative artwork of Ref.^33^ to calculate the visual appeal score and its relative importance. Evaluators compare and rank artworks based on the abovementioned characteristics using the ranking and relative importance procedures applied in Steps 7 and 8 using the FTOPSIS evaluation technique. The score can aid in establishing a relative measure of importance for qualities and provide the visual appeal score of the corresponding artwork. With the help of an analysis of the ranking procedure, the attributes of the artwork alternatives were evaluated using FTOPSIS, which helps artists choose materials and aesthetics related to the performance score and engage the audience in the environment of artwork design. Among all other existing approaches, the proposed EADF algorithm performs well by utilizing FTOPSIS and enhancing the artificial input variables related to the criteria.

**Table 1:** Comparative analysis of the EADF model with other models.

Input variables	FAHP-TOPSIS	H4MADM	FCET-CM	EADF
Color	62.52	47.16	59.66	83.74
Composition	48.12	60.42	63.11	82.37
Emotion	62.54	61.23	63.89	85.61
Contrast	58.73	66.32	74.95	97.52
Clarity	66.52	75.91	77.51	98.16
Harmony	70.18	79.56	81.74	95.49
Sensitivity	65.16	75.14	82.43	96.44

The sensitivity analysis shown in Table 1 is an essential process in multiple criteria decision-making (MCDM) for assessing the dependability and resilience of a selected decision-making approach. The method evaluates how alterations in input parameters or criteria weights impact the ultimate conclusion or ranking of options. Based on the research design and goals, sensitivity analysis can be performed at several points in a study. Some studies have conducted sensitivity analyses after presenting the main results to offer insights into the stability and consistency of the decision-making process. The timing is contingent on the intricacy of the issue, data accessibility, and research objectives. Integrating sensitivity analysis enhances the reliability and validity of decision results.

The environmental art design framework is an innovative method that incorporates the fuzzy technique for order preference by similarity to the ideal solution in assessing environmental art design practices. The methodology evaluates artworks by considering criteria such as audience involvement, environmental impact ratio, sustainability analysis, and visual appeal score, offering a thorough assessment of the artwork. The EADF presents a systematic and quantitative way to assess artworks by considering their aesthetic appeal, environmental effect, and sustainability. The platform provides a thorough assessment framework that considers the intricate relationships among artistic expression, technical advancements, and ecological factors. The EADF highlights how environmental awareness is becoming increasingly important in modern art, focusing on sustainability, resilience, and lasting effects. This study concludes that the EADF surpasses existing algorithms such as FAHP-TOPSIS, H4MADM, and FCET-CM in terms of comprehensiveness and flexibility. The EADF incorporates many evaluation criteria and offers a detailed understanding of environmental art design techniques. The EADF utilizes FTOPSIS to provide a versatile and robust method for decision-making that accommodates many viewpoints and preferences. The EADF emphasizes the significance of incorporating environmental factors into art and design techniques, which enriches the discussion on sustainability, creativity, and innovation in the arts. Additional study and collaboration are necessary to enhance and broaden the framework, guaranteeing its relevance and applicability in a swiftly changing artistic environment.

This study highlights the significance of interdisciplinary collaboration in tackling intricate difficulties in art, technology, and environmental awareness. The text emphasizes the importance of technology in art and design by showcasing how computational methods can improve data-driven decision-making. This study highlights the increasing awareness of environmental challenges in the artistic community, the significance of thorough evaluation frameworks, and the necessity for ongoing enhancement in research and practice. This study offers vital insights into how art, technology, and environmental awareness intersect, encouraging innovation and collaboration in the creative sector. The environmental art design framework (EADF) is a novel method for incorporating environmental factors into art and design processes.

Nevertheless, it has specific constraints such as oversimplification of environmental impact, subjective evaluation, limited data scope, assumptions in the FTOPSIS technique, absence of stakeholder participation, and inadequate consideration of cultural aspects. The framework may not comprehensively encompass the complex nature of environmental sustainability, which is shaped by resource consumption, waste creation, and ecological impact. The framework may lack consideration of cultural dynamics and identity, which are essential for creating good environmental art. Further validation is needed to ensure its effectiveness.

## Conclusion

Environmental art design exists at the crossroads of artistic expression, technical innovation, and ecological practices. With increasing complexity, the proposed EADF-based FTOPSIS method has emerged as a reliable instrument. This algorithm recognizes the multifaceted character of art by considering elements such as visual appeal, environmental effects, sustainability, and community participation. It provides a planned structure that encourages those making decisions in the development process by leveraging the potential of Fuzzy TOPSIS. It analyses artworks scientifically and confronts the inherent ambiguity and subjectivity in the artistic domain. It assesses the deviation of artworks from optimal and unfavorable results by using fuzzy logic to interpret the intricacies of aesthetic worth and employing Euclidean distance measures. The effectiveness and adaptability of the EADF model are highlighted by validating it with measures such as audience interaction, environmental impact, sustainability, and visual appeal. This algorithm catalyzes the convergence of creative genius and environmental conscience, allowing practitioners to create artistically resonant places while harmonizing with nature and society. The limitations of the EADF algorithm make it possible to apply FTOPSIS with the assumption of linear correlations between attributes, which is not necessarily the case in the complex aesthetic domain. The environmental art design framework is a novel method for environmental art design that utilizes the fuzzy technique for order preference by similarity to an ideal solution approach. The framework aims to assist artists in making well-informed judgments on visual appeal, environmental effects, sustainability, and audience engagement. It has been proven to effectively reduce adverse environmental impacts and encourage the use of sustainable materials. The EADF also offers insights into the durability and environmental sustainability of creative creations. It enables artists to make well-informed decisions about color, composition, and emotional impact. The framework combines AI and machine learning to connect artistic creation with environmental awareness. Nevertheless, this approach is limited by the assumption of linear connections between qualities, thereby oversimplifying aesthetic decision-making. Future studies should concentrate on nonlinear pattern recognition and modeling attribute interactions. The application of state-of-the-art AI and machine learning algorithms in environmental design practice can assist with nonlinear patterns and attribute interactions in the future.

## Data Availability

The data used to support the findings of this study are all in the manuscript.
